# Comparative transcriptomics reveals shared gene expression changes during independent evolutionary origins of stem and hypocotyl/root tubers in *Brassica* (Brassicaceae)

**DOI:** 10.1371/journal.pone.0197166

**Published:** 2018-06-01

**Authors:** David J. Hearn, Patrick O’Brien, Sylvie M. Poulsen

**Affiliations:** Department of Biological Sciences, Towson University, Towson, Maryland, United States of America; Instituto de Biologia Molecular y Celular de Plantas, SPAIN

## Abstract

Plant succulence provides a classic example of evolutionary convergence in over 40 plant families. If evolutionary parallelism is in fact responsible for separate evolutionary origins of expanded storage tissues in stems, hypocotyls, and roots, we expect similar gene expression profiles in stem and hypocotyl / root tubers. We analyzed RNA-Seq transcript abundance patterns in stem and hypocotyl / root tubers of the *Brassica* crops kohlrabi (*B*. *oleracea*) and turnip (*B*. *rapa*) and compared their transcript expression profiles to those in the conspecific thin-stemmed and thin-rooted crops flowering kale and pak choi, respectively. Across these four cultivars, 38,192 expressed gene loci were identified. Of the 3,709 differentially-expressed genes (DEGs) in the turnip: pak choi comparison and the 6,521 DEGs in the kohlrabi: kale comparison, turnips and kohlrabies share a statistically disproportionate overlap of 841 DEG homologs in their tubers (*p* value < 1e-10). This overlapping set is statistically enriched in biochemical functions that are also associated with tuber induction in potatoes and sweet potatoes: sucrose metabolism, lipoxygenases, auxin metabolism, and meristem development. These shared expression profiles in tuberous stems and root / hypocotyls in *Brassica* suggest parallel employment of shared molecular genetic pathways during the evolution of tubers in stems, hypocotyls and roots of *Brassica* crops and more widely in other tuberous plants as well.

## Introduction

**Fig 1:**
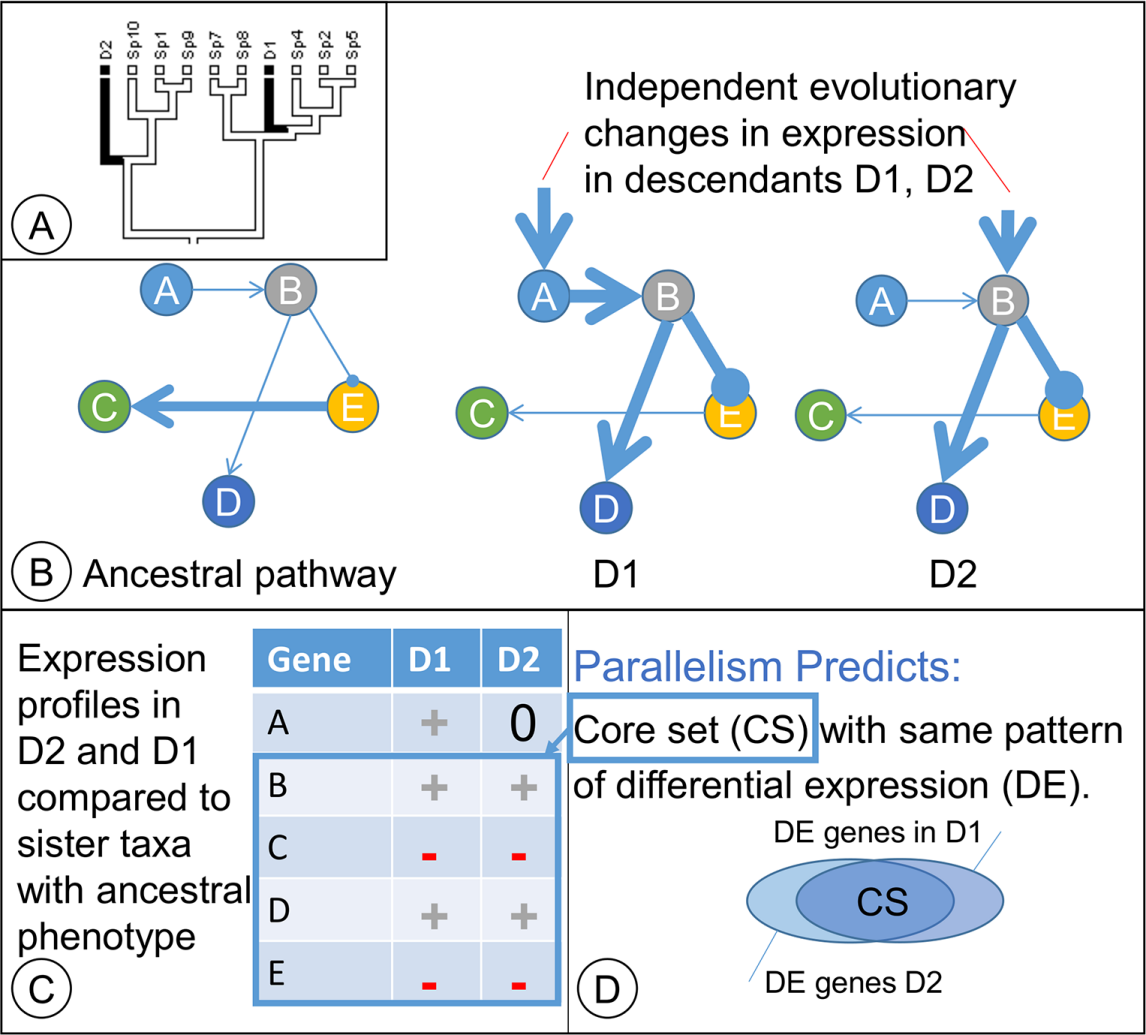
Parallelism profiling. (A) In a hypothetical example, a phylogeny with descendant taxa D1 and D2 have convergent phenotypes (black). (B) Genetic regulatory networks to illustrate consequences of evolutionary changes in expression to gene A in D1 and separately to gene B in D2; both changes result in the black phenotype. (C) Changes in gene expression relative to taxa with the common ancestor’s phenotype. (D) Descendants D1 and D2 have a “core set” of differentially expressed genes in common.

Parallel evolutionary processes provide key entry points into the analysis and discovery of core genes that contribute to the recurrent evolution of similar phenotypes. As classically defined, evolutionary parallelism arises when separate lineages evolve similar phenotypes through parallel changes in the expression of homologous genes. In contrast, evolutionary convergence results in similar phenotypes through the actions of different genes in often distantly related lineages. Arendt and Reznick [1] suggested that a more accurate picture presents a spectrum that spans these extremes, as the evolution of similar, complex phenotypes in diverse taxa may involve both shared and novel components in a network of gene interactions in which what is shared and what is novel may be difficult to disentangle in practice [2]. Thus, the analysis of evolutionary parallelism might be refined to discover those shared, core gene homologs involved in the evolution and development of convergent phenotypes as well as the sets of novel genetic pathways in specific lineages (Fig 1).

Fig 1 illustrates how separate evolutionary events can result in parallel shifts in gene expression during the evolution of a character with two states. The ancestral white state has shifted to a black state in two descendants (D1 and D2) through parallel evolutionary processes (Fig 1A). In the direct ancestor of D1, a mutation in regulatory gene A caused its upregulation, and the cascade of downstream changes resulted in the black phenotype (Fig 1B). In D2, an independent evolutionary event in gene B caused a similar cascade of events in downstream genes that parallel changes in expression of homologous genes in D1 (Fig 1B). Compared to a taxon from a sister clade with the ancestral phenotype, D1 and D2 show parallel shifts in expression pattern in the downstream genes (Fig 1C) even though the initial evolutionary changes occurred in non-homologous genes (e.g., changes to gene A vs. gene B). The result is a partial overlap in genes that show changes in expression, with a ‘core set’ of homologous genes showing similar changes in gene expression in D1 and D2 compared to the ancestral phenotype (Fig 1D).

Two questions naturally follow from this framework: (question 1) which core gene homologs show parallel evolutionary changes in gene expression patterns, and (question 2) are there more gene homologs with parallel shifts in expression that expected by chance? A statistically disproportionate number of homologs would be expected to have similar shifts in gene expression during parallel evolutionary events. In contrast, if convergent evolutionary processes were largely driven by separate, independent molecular genetic pathways, some gene homologs may still evolve similar shifts in expression, but these shared shifts would be coincidental and no different in number than expected by chance.

Analyses of RNA-Seq [3] transcript abundance profiles in model and non-model organisms now provide a route to answer these two questions at a genome-wide scale. We analyzed exomes from separate evolutionary origins of parenchymatous woods in tuberous hypocotyls, roots and shoots of *Brassica*. We identified the core genes (question 1) and evaluated the statistical significance of the size of this core set (question 2).

### Parenchymatous woods

**Fig 2:**
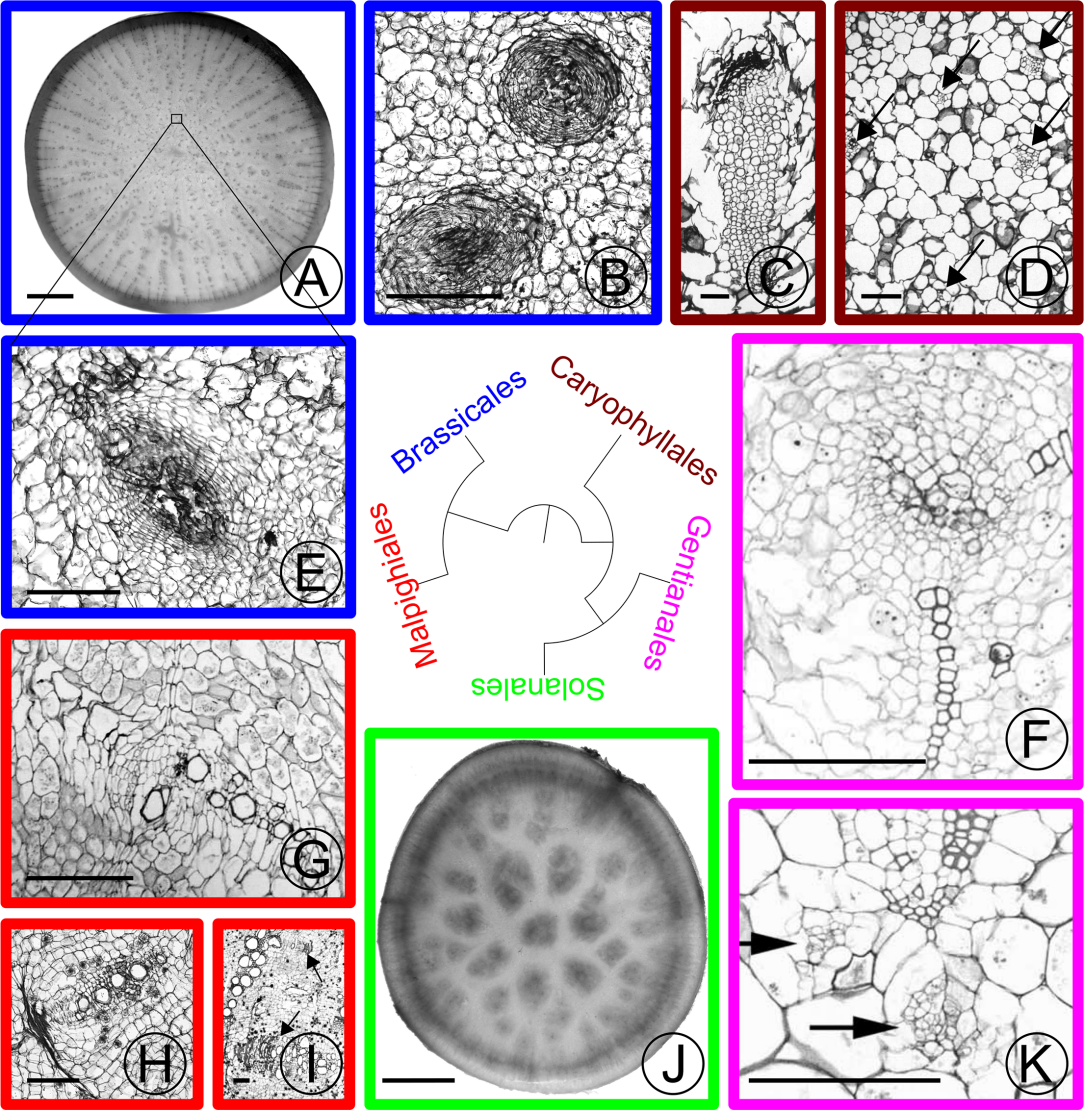
Stem and hypocotyl / root vascular bundles in parenchymatous tissues of various eudicot taxa. Taxonomic orders are colored based on the label in the phylogeny. (A) Turnip (*Brassica rapa rapa*) cross section. Small dots in the center of the section are supplemental vascular bundles (SVBs). (B) Close-up of medullary SVB in the stem of kohlrabi (*Brassica oleracea gongylodes* cv. ‘Purple Vienna’). A small zone of xylem encircles crushed phloem in the amphivasal bundle arrangement. (C) Close-up of old medullary SVB from the trunk of *Trichocereus chilensis* (Cactaceae) showing a long tail of xylem adjacent to phloem that is capped with crushed secondary phloem (dark band). (D) Cross section of young stem just below the shoot apical meristem of *Subpilocereus ottoni* (Cactaceae) showing the distribution of five medullary bundles (arrows). (E) Close-up of SVB of turnip from boxed region in (A), showing the same arrangement of vascular tissues as kohlrabi. (F) Older medullary SVB from stem of *Pachypodium namaquanum* (Apocynaceae) with two zones of xylem and a zone of phloem interior to the xylem. (G) Old collateral medullary SVB from stem of *Adenia keramanthus* (Passifloraceae). (H) Old collateral SVB from root tuber of *A*. *inermis*. (I) Large collateral medullary SVBs from stem of *A*. *metamorpha*. (J) Cross section through tuberous root of *Ipomoea batatas* (Convolvulaceae). Dark regions in the center of the root are zones of proliferation with SVBs. (K) Medullary bundles from *Caralluma burchardii* (Apocynaceae) with two medullary phloem bundles (arrows) below protoxylem. Scale: (A), (J) 10mm; (B)-(I),(K): 250 m. (C) and (D) adapted from [4] with permission from the author; (F) and (K) adapted from [5] with permission from the author.

Woods in which parenchyma storage cells predominate by volume are referred to as parenchymatous woods (PWs) which occur in hypocotyls, roots and shoots. PWs evolved independently in at least 50 genera (see [4–5], Fig 2; [6]). Here, we refer to stem, hypocotyl, or root structures with extensive PW collectively as tubers. In turnip, the hypocotyl and root both contribute to tuber formation, so these tubers are referred to as hypocotyl / root tubers. Two developmental events highlight the uniqueness of PWs. Additional meristems that are separate from the secondary vascular cambium (VC) are responsible for these distinct features of PW: (1) the proliferation of parenchyma (PP) through additional cell divisions and (2) the differentiation of supplemental vascular bundles (SVBs) in the storage tissue [4,5,7,8]. Additionally, at the VC itself, taxa with PWs exhibit a dramatic evolutionary shift from the lignified xylem of hard-wooded ancestors to xylem composed almost entirely of relatively undifferentiated storage parenchyma (e.g. [9]).

Food crops such as parsnips, turnips, carrots, beets, and others develop extensive PW. PWs are also of fundamental ecological importance in arid regions where they can store water and modulate stem osmotic potential [6,10,11]. ‘Charismatic megaflora’ [6] such as arid-adapted cacti and baobabs provide quintessential examples of PW. Therefore, study of PWs will elucidate fundamental mechanisms of plant development, will foster a greater understanding of the evolution and development of important food crops, and will reveal mechanisms of adaptation to water- and heat-stressed environments.

### Evolutionary processes responsible for PWs

Although PWs in roots, shoots, or hypocotyls represent a complex phenotype, several lines of evidence suggest that their evolution may proceed through parallel, switch-like mechanisms. First, phylogenetic comparative studies in *Adenia* (Passifloraceae) [12–14] and more broadly throughout the eudicot plants [6] indicate that taxa with tubers in stems are more closely related to taxa with tubers in hypocotyl / roots than expected by chance. This pattern prompted the hypothesis that spatial switches in the expression of shared developmental modules led to the evolution of PWs in both shoots and roots [6,7,13]. Wood storage characters in the genus *Adenia* evolve as a module that is semi-dissociable from other wood developmental modules [14]; this modularity was proposed to account for the evolutionary lability of PW evolution. Moreover, dramatic transitions in the abundance of PW can occur on time scales of fewer than one million years [13]. Lastly, the relatively specialized features of PW, such as PP and SVBs, appear to be correlates of PW evolutionary across a diversity of taxa [5,7]. Cell proliferation and SVBs occur in the PWs of Cactaceae [4,8], Apocynaceae [5], *Pelargonium* (proliferation; [15]), *Adenia* [7,12], sweet potatoes [16], carrots [9], and turnips [17]. Hearn [7], Mauseth and Sajeva [8], Carlquist [18] (for succulent stems and roots) and Ogburn and Edwards [19] (for succulent leaves) suggested that innervation of succulent plant organs by VBs is important for transport in otherwise diffusion–limited tissues. Collectively, these comparative lines of evidence hint at shared mechanisms of PW evolution and development.

### PW development

Because of their agronomic interest, extensive research is beginning to identify the genes responsible for the induction and development of storage organs in multiple crops: potato [20,21], sweet potato [16,22–26], cassava [27], Jerusalem artichoke [28,29], carrot [9], radish [30,31], turnip [32–35] and other taxa (reviewed by [36]). No studies have investigated exomes of tubers in above-ground stems. In tubers of previously-investigated taxa, the vascular cambium produces little lignified tissue, proliferation of parenchyma usually occurs in the centers of these organs [16,17,37,38], and SVBs are often present, especially in older tubers [16,17]. This literature highlights key participants in tuber development, including the actions of the plant hormones gibberellic acid (GA), jasmonic acid (JA), abscisic acid (ABA), brassinosteroids (BA) and auxins (reviewed by [39]). Sucrose production and lipoxygenases [20] also play central roles during tuber induction. In both turnips [34] and potatoes [40], cessation of exposure to GA induces stem tuber formation. Sucrose, JA, and ABA appear to be positive regulators of tuber growth in potatoes [40]. Indeed, tuber formation ceases below a threshold sucrose concentration in potatoes [40]. The actions of auxins are more complex [21], but it is clear that auxins and HD-ZIP III transcription factors play a key role in vascular differentiation (e.g., [41,42]) that may also occur in tubers.

If parallelism is responsible for the separate evolutionary origins of tubers in diverse taxa, then it is expected that genes participating in the above functions will be enriched in the set of homologous, differentially expressed genes (DEGs) in tubers of different taxa. Moreover, it is expected that genes responsible for cellular proliferation, meristem development, and xylem differentiation (in the SVBs) will be enriched in the parenchymatous tissues of tubers relative to the parenchymatous tissues of close relatives that lack PWs.

### Hypotheses

The genus *Brassica* L. (Brassicaceae) is uniquely poised for PW research. PW evolved at least three times in stems of kohlrabi (*B*. *oleracea gongylodes*) and in hypocotyl / roots of turnips (*B*. *rapa rapa*) and rutabagas (*B*. *napus napobrassica*). We carried out an RNA-Seq experiment in *Brassica* in which we compared gene expression profiles between two pairs of taxa with contrasting PW phenotypes. In the first pair, we compared gene expression profiles of kohlrabi (with a stem tuber) to those of a narrow cane-forming flowering kale (*B*. *oleracea acephala*). In the second pair, we compared gene expression profiles of turnip to those of pak choi (*B*. *rapa chinensis*). We expected to find (1) a core set (see Fig 1) of genes with similar shifts in expression in kohlrabi and turnips (relative to kale and pak choi, respectively) that is statistically larger than expected by chance and (2) enrichment in this ‘core set’ of gene functional classes that contribute to tuber development in other taxa (e.g., sucrose biosynthesis, lipoxygenases, and genes involved in plant hormone regulation).

## Methods

### Study organisms

**Fig 3:**
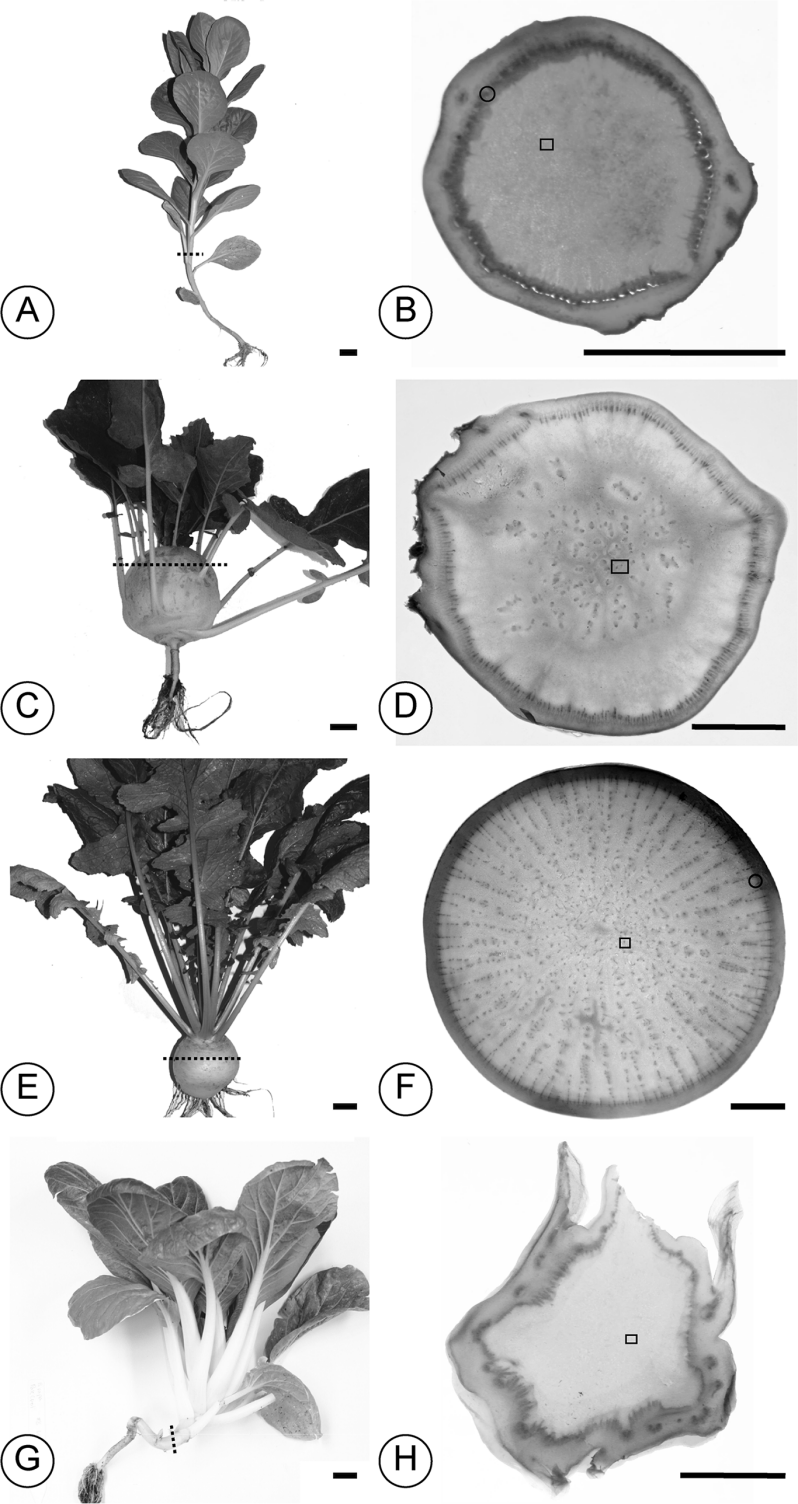
***Brassica* plant form** (A)–(B) Flowering kale (*Brassica oleracea acephala* cv. ‘White Crane’. (C)–(D) Kohlrabi (*B*. *oleracea gongylodes* cv. ‘Express Forcer’). (E)–(F) Turnip (*B*. *rapa rapa* cv. ‘Tokyo Hybrid’). (G)–(H) Pak choi (*B*. *rapa chinensis*). In each set of two panels, the first panel presents the plant form at harvest. Dotted lines represent where the cross section presented in the second panel was made. The second panel presents an overview of the cross section. The specks in panels (D) and (F) are the SVBs that are absent in flowering kale (B) and pak choi (H). Black spots in the cortices of (B) and (H) are leaf vascular traces. Scale bars: (A), (C), (E), (G): 2.5 cm; (B), (D), (F), (H): 10mm.

Cultivars of *Brassica* (Brassicaceae) were selected that contrasted in the volume of parenchymatous tissue. In *Brassica oleracea*, kohlrabies (*B*. *oleracea gongylodes*) initiate PW development at or above the fifth stem node [43] and develop a globose parenchymatized tuber. The hypocotyl and lower stem develop hard, lignified wood. The kohlrabi cultivar (cv.) ‘Express Forcer’ was selected due to the large size of its tuber and its fast time to maturity. Anecdotally, kohlrabies first appeared ~500 years ago [44] and represent a very recent crop of European origin. In contrast, kales (*B*. *oleracea acephala*) exhibit closer to the ancestral form of *B*. *oleracea* and are known from the gardens of Greek antiquity. Although members of the same species, kales exhibit more lignified woods compared to the parenchymatous wood of kohlrabies, and they have longer stems in general. The kale cv. ‘White Crane’ was sampled for its long, narrow stem. Fig 3 illustrates the selected *Brassica* cultivars’ forms and gross anatomy (hand sections were stained using a modified Shabman staining series [45]).

In *Brassica rapa*, taxon selection paralleled those of *B*. *oleracea*. The turnip (*Brassica rapa rapa*) cv. ‘Tokyo Cross’ was selected for its expanded hypocotyl / root storage wood, and pak choi (Meyer Seed Company, Baltimore; *B*. *rapa chinensis*) was selected for its relatively long, narrow stem and hypocotyl.

Twenty plants of each cultivar were grown from seed in Sunshine Universal Mix No. 1 (SunGro Horticulture) soil under ambient light and 21°C-43°C temperature in the Towson University greenhouse facility. Plants were randomly placed on greenhouse benches to avoid positional effects. Plants were watered daily in the morning, and fertilizer (Scotts Miracle-Gro ®) was applied weekly along with neem / pyrethrin sprays (Bonide) to reduce greenhouse pests. Ten of the twenty individuals were randomly selected and harvested from each cultivar for RNA extraction and exome sequencing. Plants were harvested after three months in midday following a morning watering. At harvest, tubers were actively developing in turnips and kohlrabi, so we expected to find the developmental events of interest in the storage parenchyma (PP, SVB development). Pak choi individuals were harvested earlier, after 2 months, as inflorescences were initiated prior to three months in pak choi.

### Anatomy

Appropriate sampling of developmental stages was important to assure that similar developmental events in PW were being compared. Previous studies [7] observed that at any given point in time during PW development, PP and VB differentiation are occurring simultaneously at different stages of development in multiple regions of the tuber. So, one can sample across these PW developmental events by sampling tissues from actively-developing, older turnip and kohlrabi tubers. Unlike many other studies (e.g., [16,24,27,32,39]), the goal here was not to investigate tuber induction. Rather, it was to investigate exome expression among separate evolutionary origins of SVBs and PP. We expected no (or less) cell proliferation and SVBs in the narrow regions of stem and hypocotyl / root parenchyma in kale and pak choi. Such parenchyma would provide a natural backdrop against which to compare tissues from kohlrabi and turnip in which developmental events were ongoing. Thus, we expected the DEGs in turnip and kohlrabi storage parenchyma, when compared to pak choi and flowering kale, respectively, to represent genes responsible for proliferation of parenchyma and differentiation of SVBs compared to processes involved in the maintenance of parenchyma alone.

Namikawa [17] previously established that cellular proliferation and SVBs occur in turnip storage organs. Namikawa’s [17] studies in turnip illustrated the break-up of xylem as PP disrupted the normal patterning of xylem. Relatively little anatomical characterization has been carried out in kohlrabi [43]. In [43] and [17] the resolution of images was inadequate to characterize the detailed patterning of SVBs, and it was unclear whether VBs occurred in the storage stems of kohlrabi.

Prior to RNA-Seq analyses, we first established that PP and SVB development occur in the selected turnip and kohlrabi cultivars and that they were absent or rare in conspecifics that lack storage wood. Two of the ten plants that were harvested for RNA were also sampled for anatomical analysis. Stem and hypocotyl tissues were fixed in FAA (formalin : ethanol : glacial acetic acid : water = 2:10:1:7). These tissues were infiltrated and embedded in paraffin according to the ethanol dehydration series of Ruzin [46] and sectioned to <20μm on a sliding American Optical 820 microtome. Sections were stained using the Shabman staining series [45] that highlights cambial regions, and they were mounted on slides using Cytoseal 60 (Thermo Scientific). Longitudinal and transverse sections were made through the thickest portions of the stem and hypocotyl region of each cultivar.

### Tissues for exome analysis and sequencing

Tissue from the 1 cm core from centers of stems (kale, kohlrabi) or from hypocotyl (turnip, pak choi) were sampled at the widest points via hand dissection. Root tissue in pak choi was highly lignified, so it does not provide an appropriate tissue comparison to the parenchyma in turnip root tubers, so parenchymatized hypocotyl tissue at the interface with the stem was sampled. Tissues were flash frozen in liquid nitrogen for total RNA extraction using the MoBio RNA kit with on column DNAse treatment (MoBio 13550). Total RNAs from the ten pooled biological replicates of each cultivar were sent to the University of Missouri, Columbia, DNA Core sequencing facility for Illumina TruSeq library preparation and sequencing on the Illumina HiSeq 2000 platform. Library prep followed the proprietary TruSeq Kit unstranded protocol v. 2. Two HiSeq 2000 (v3) lanes of 100 base pair (bp) single end reads were run with two cultivars multiplexed per lane.

### Exome assembly and expression

Sequence reads were trimmed to remove low quality sequences from their ends using Trimmomatic [47], and trimmed read quality was further checked by fastqc (Babraham Bioinformatics). As the genome of *B*. *rapa* cv. ‘Chiifu’ is publicly available with large scaffolds [48], a referenced-guided assembly of the reads used the *B*. *rapa* (v. 1.2) genome available from BRAD [49] and additional sequence information from Phytozome [50].

Assembly was carried out using the Tophat (v1.3.2) [51], Bowtie (version 0.12.9) [52], Cufflinks (v2.0.2) [53] pipeline. Cuffmerge, part of the Cufflinks package, labels transcripts from different samples according to a previous annotation of the reference genome so that the locus identity of the Cufflinks transcripts is known across samples. Cufflinks quantifies relative transcript abundance levels using the fragments per kilobase of exon per million fragments mapped (FPKM) metric, which is similar to the RPKM metric [54].

To evaluate how well *B*. *oleracea* paralogs were assembled using the *B*. *rapa* genome as a reference, we carried out a gene tree analysis of *PLETHORA* (*PLT*; *AINTEGUMENTA LIKE*) and HD-ZIP III *HOMEOBOX* (*HB*) gene families to check that assembled transcripts cluster with their previously annotated paralogs. We expected assembled transcripts from *B*. *oleracea* to be most closely related to previously annotated genes from *B*. *oleracea*. *Brassica oleracea* and *B*. *rapa* gene copies were expected to be sister to one another and the *Brassica* gene copies to be sister to the homologous *Arabidopsis* gene. Assembled transcript sequences and previously annotated sequences were aligned using MUSCLE [55]. Analyses for each gene family were carried out using RAxML [56] with 1000 random addition sequence bootstrap replicates.

Cuffdiff (also part of Cufflinks) tested for differences in transcript abundance by modeling variation in transcript abundance in a manner that accounts for isoforms; Cuffdiff uses a null hypothesis of no difference in transcript abundance between samples [57]. The DEGs were determined at an α significance level of 0.05 corrected for multiple tests. Once the DEGs were identified for the turnip : pak choi comparison and for the kohlrabi : flowering kale comparison, we tested whether the overlap between these two sets of DEGs was statistically significant. We expected this core set (see Fig 1) of jointly differentially expressed genes to be statistically larger than expected by chance.

To formalize this statistical test, we considered the overlap among three sets of genes. One set, Ω, of size *N* contains all expressed genes, a second set, K_1_, of size *k*_*1*_ contains all the DEGs found in the comparison between turnip and pak choi exomes, and the third set, K_2_, contains all the DEGs found in the comparison between kohlrabi and flowering kale exomes. Under the null hypothesis of pathway independence for tuber development, the set K_2_ is a random sample from Ω with respect to K_1_. In other words, any overlap between K_1_ and K_2_ is due to sampling the same gene homologs by chance from the set of all expressed genes. This is a similar null model used by other studies of differential expression [58] and is implemented in the R package GeneOverlap [59]. Under the null hypothesis, the size of the core set, *r*, is hypergeometrically distributed [58,59].

The direction of change in expression also has to be accounted for. As a close approximation for large samples of genes, this doubles the size of the sample space Ω as each DEG can be either up-regulated or down-regulated. So, the probability by chance of finding *r* genes that are differentially expressed in both kohlrabi and turnip *and* that share the same direction of differential expression (e.g., up-regulated in both K_1_ and K_2_) is:

$$P(r|N,k_{1},k_{2})=\frac{\left(\begin{array}{c} k_{1}\\ r \end{array})(\begin{array}{c} {2N-k}_{1}\\ k_{2}-r \end{array}\right)}{\left(\begin{array}{c} 2N\\ k_{2} \end{array}\right)}$$ [Eq 1]

Due to the exchangeability of the hypergeometric distribution, *k*_*1*_ and *k*_*2*_ can be swapped with no change in the probability. The *p* value associated with an observed *r* is:

$$P(ror\;more\;shared\;DEGs)=\sum_{r_{i}=r}^{r_{i}=k_{1}}\frac{\left(\begin{array}{c} k_{1}\\ r_{i} \end{array})(\begin{array}{c} {2N-k}_{1}\\ k_{2}-r_{i} \end{array}\right)}{\left(\begin{array}{c} 2N\\ k_{2} \end{array}\right)}$$ [Eq 2]

At the standard significance level of α = 0.05, one can conclude that the size of the core set is significantly greater than expected by chance when the above sum is less than or equal to 0.05. This *p* value can be calculated using the R statistical package [60]:

$$p\;value=sum\left(dhyper\left(r:k_{1},k_{1},2*N‑k_{1},k_{2}\right)\right).$$

### Functional enrichment tests

An enrichment test compares two sets of genes to determine if one of the sets has disproportionately more genes of a functional class than the other set. To test for enrichment in the core set, we compared gene functions of loci in the core set to the gene functions of loci in a reference set of 10,000 randomly selected loci from the set of all expressed loci. For each of these loci, we associated Gene Ontology (GO) terms. Some GO terms were available from Phytozome and BRAD, but we found that these annotations were for broad functional classes and did not provide detailed functional information. So, we used the Blast2GO [61] annotation pipeline to associate GO terms with these loci. We used false discovery rate (FDR)-adjusted [62,63] Fisher’s Exact Test to find which GO categories had disproportionately more (or disproportionately fewer) loci in the core set compared to the reference set.

Using a parallel approach, we also tested for the enrichment of KEGG pathway [64] enzymes in the core set by comparing the number of genes in each KEGG pathway between the core set and the reference set to test the null hypothesis that the frequencies of genes in each KEGG path way is no different between the core and reference sets. In particular, we predicted disproportionately more sucrose and starch biosynthesis pathway genes in the core set based on the role of sugars in tuber induction in potato [40].

## Results and discussion

### Anatomy

**Fig 4:**
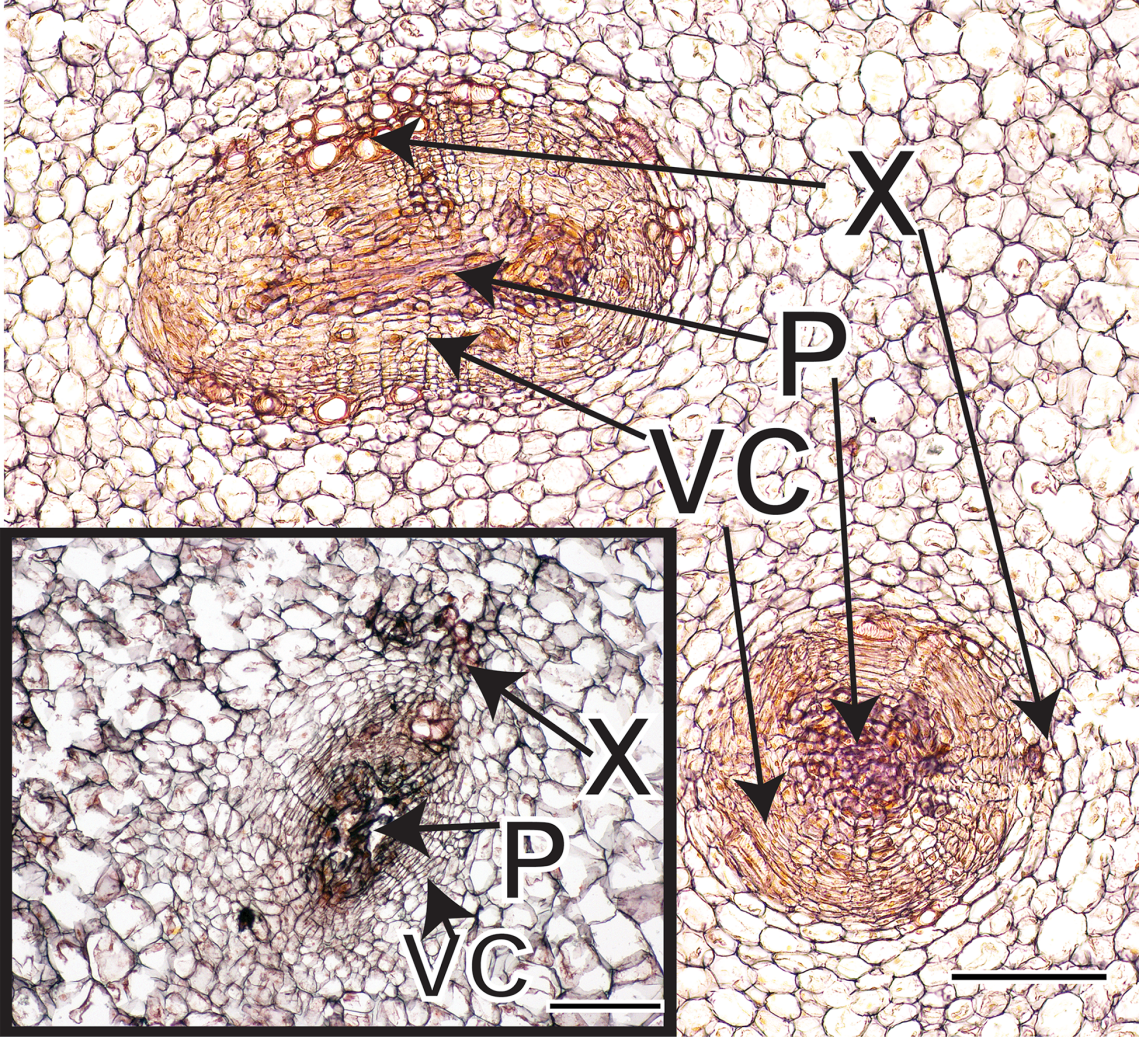
Cross section of amphivasal VBs in kohlrabi and turnip (inset). VBs are longitudinally-oriented with xylem surrounding a vascular cambium that produces phloem internally. Vascular bundles occur in the centers of stems or hypocotyl / roots as indicated by the small rectangles in Fig 3D, and Fig 3F. Scale bars: 100μm. X = xylem; P = phloem; VC = vascular cambium.

**Fig 5:**
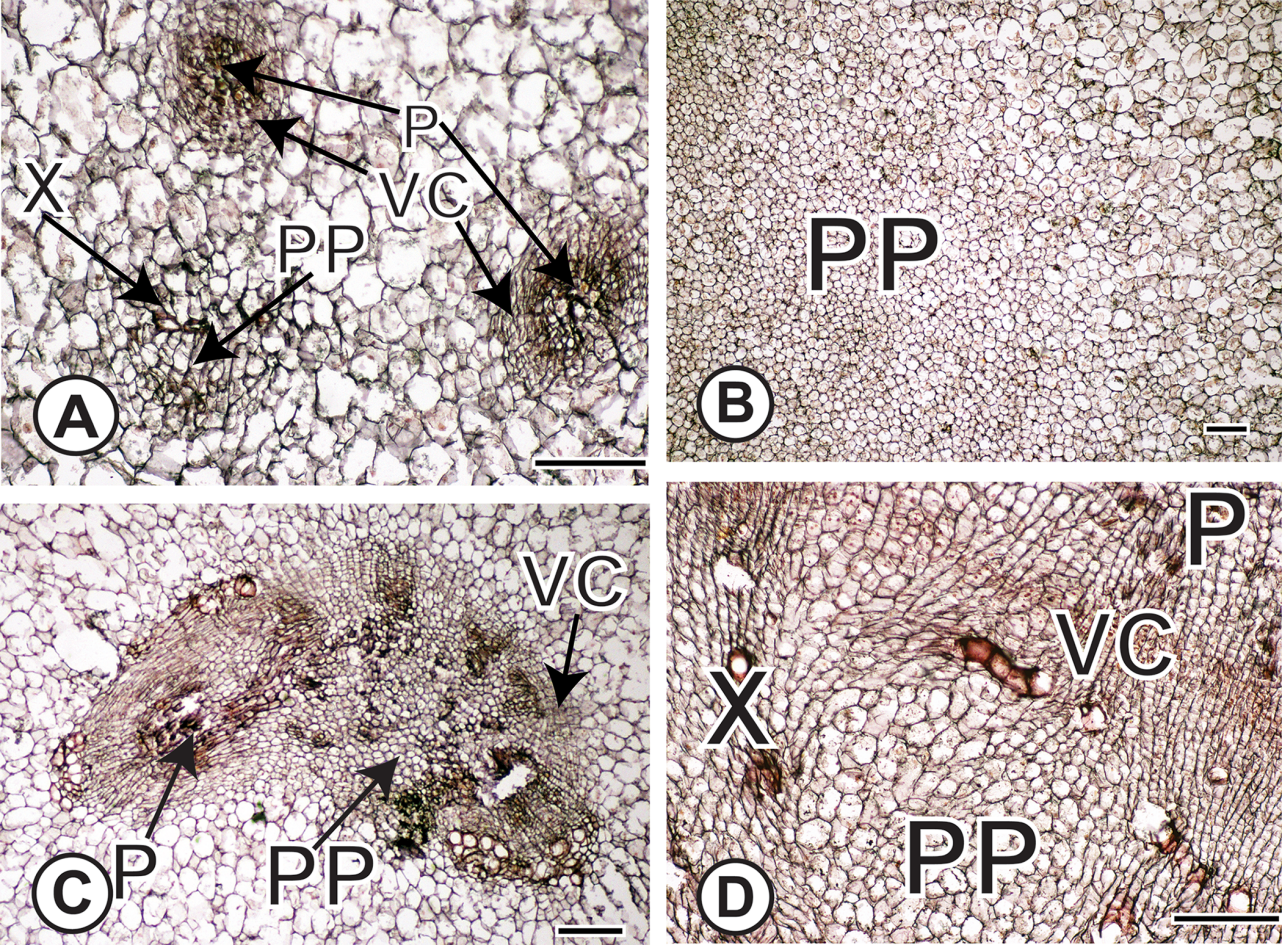
Anatomical evidence of proliferation of parenchyma (PP). (A) PP within a VB in turnip in which proliferative cells are surrounded by scattered vessel elements. Two mature vascular bundles are labeled as well. (B) PP in center of kohlrabi stem. Note the profusion of smaller proliferative cells next to the larger, mature parenchyma cells. (C) PP has disrupted the patterning of a SVB. Note the fragments of phloem (darker staining) near the lighter-staining, small proliferative cells. (D) Intrusive proliferation of parenchyma near the main vascular cambium results in distorted cells and disrupted vessel elements. Note the sheared parenchyma cells next to the red-stained vessel elements that are no longer longitudinally oriented. From circled region in Fig 3F. Scale bars: 100μm. X = xylem; P = phloem; PP = proliferation of parenchyma; VC = vascular cambium.

Amphivasal VBs were present in centers of kohlrabi cv. ‘Express Forcer’ storage tissue at a density of ~0.58 mm^-2^ and in turnips cv. ‘Tokyo Cross’ at a density of ~1.43 mm^-2^ (Fig 4). Proliferative parenchyma was inferred from tissue sections based on three anatomical markers of proliferative parenchyma [12]: distortion of cell shape in cells surrounding a zone of intrusive proliferative growth, clusters of smaller cells at centers of zones of proliferation, and breakup of surrounding tissue patterns in zones of proliferation (Fig 5).

**Fig 6:**
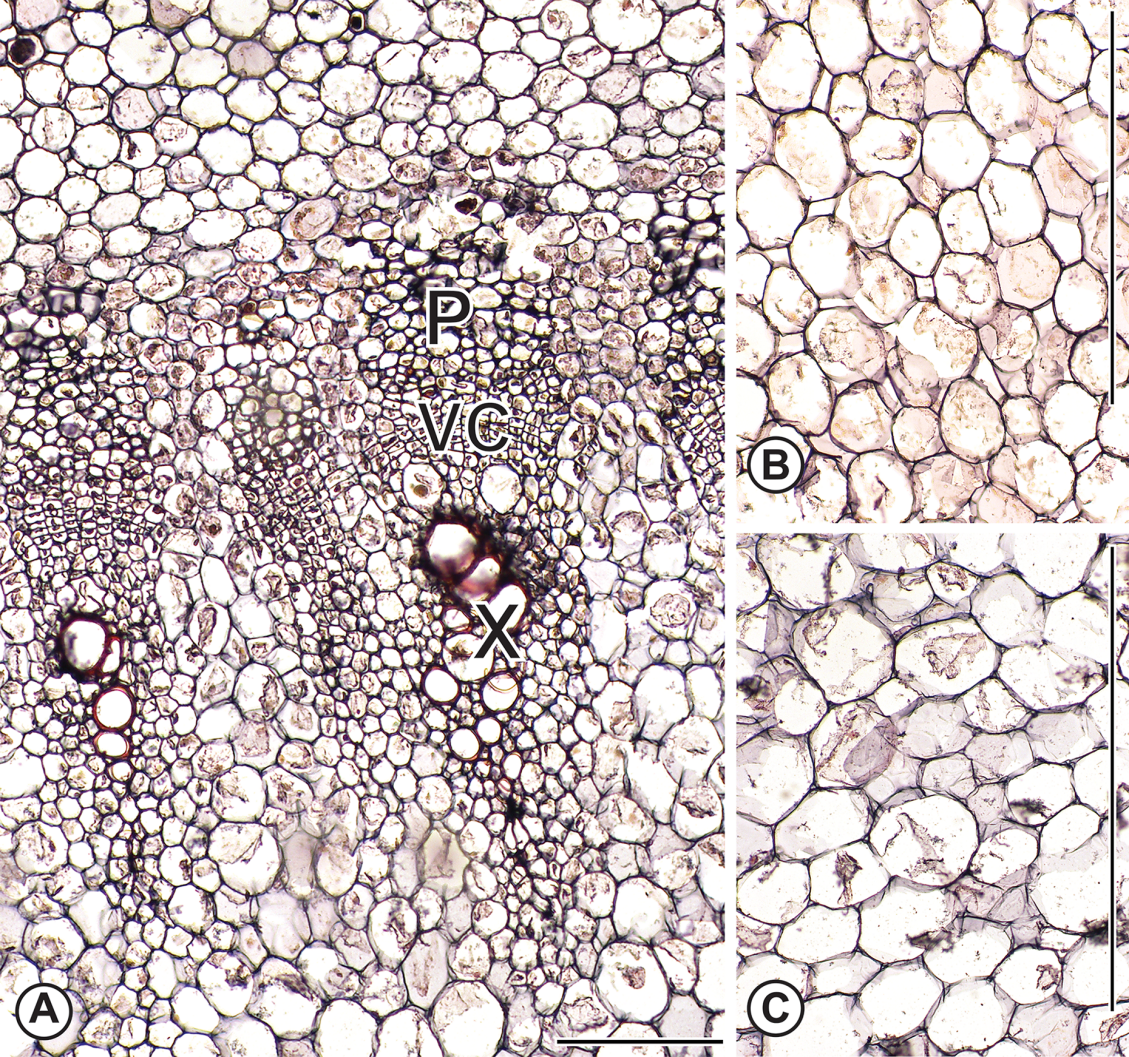
Anatomy of flowering kale and pak choi. (A) Flowering kale vascular cambium and associated secondary xylem and phloem in flowering kale stem corresponding to the region with the small circle in Fig 3B. No intrusive growth is visible and cells are nearly isodiametric in contrast to the sheared cells near the turnip vascular cambium (Fig 5D). (B) and (C) Parenchyma cells in the vicinity of the small rectangles of Fig 3B and 3H of flowering kale and pak choi, respectively. Scale: A: 100μm; B, C: 400μm. X = xylem; P = phloem; VC = vascular cambium.

We examined the entire transverse sections from two biological replicates of kale cv. ‘White Crane’ and pak choi (Meyer Seed Company, Baltimore) and found neither medullary SVBs nor PP (Fig 6). However, in kale, we found two to three cortical SVBs. These cortical bundles were not part of the tissue sample used for RNA analyses. Centers of kale stems were pure parenchyma, or in the case of pak choi, the hypocotyl/stem transition that was sampled was pure parenchyma.

### Exome assembly and gene expression

Assembled exomes ranged in size between 39 million bp (flowering kale) and 54 million bp (pak choi). In general, the number of assembled transcripts, the lengths of transcripts and their GC content resembled those metrics of the sequenced *B*. *rapa* genome (Table 1). The assemblies of *B*. *oleracea* had more assembled transcripts and shorter transcripts than did the assemblies for *B*. *rapa*. These differences between species may in part be due to mis-assembly of reads, but we found that Cuffmerge tended to merge these smaller transcript fragments into the full length transcript (S1 Data presents the transcripts produced by Cuffmerge in the GTF format). Additionally, comparisons were only between members of the same species. Assemblies for cultivars of the same species were very similar suggesting a consistency of exome structure within the species (Table 1; Sequence Read Archive (SRA) [65] project SRP144262) Assembly statistics in Table 1 are defined elsewhere [66]. In total, 7,043 Cuffmerge transcripts did not correspond directly to previously recognized *Brassica rapa* coding regions (XLOC labels for loci in S1 Table). These are worth exploring in future work, as they may represent previously unannotated loci, but they were not explored here.

**Table 1:** Assembly statistics for the four cultivars.

Cultivar	SRA accession	Number of reads	Exome size	Transcripts	Average length	Median length	Maximum length	Minimum length	N50
*Brassica rapa* genome v. 1.2	NA	NA	48237843	41173	1172	981	16227	150	1482
Flowering kale cv. 'White Crane'	SRX4020273	58423701	38832482	43520	892	658	15440	33	1320
Kohlrabi cv. 'Express Forcer'	SRX4020274	81188779	41036632	45318	906	679	16249	34	1315
Pak Choi	SRX4020272	88362643	54288367	36864	1473	1220	16633	100	1901
Turnip cv. 'Tokyo Hybrid'	SRX4020271	60949535	53529238	38568	1388	1157	16583	100	1792
Cuffmerge of the four cultivars	NA	NA	65632843	48185	1362	1063	16599	34	1876

‘Number of reads’ column presents the number of Illumina single end 100 bp reads used to generate the assembly, giving ~100-200X average coverage of the exome. Transcript lengths are in bp.

**Fig 7:**
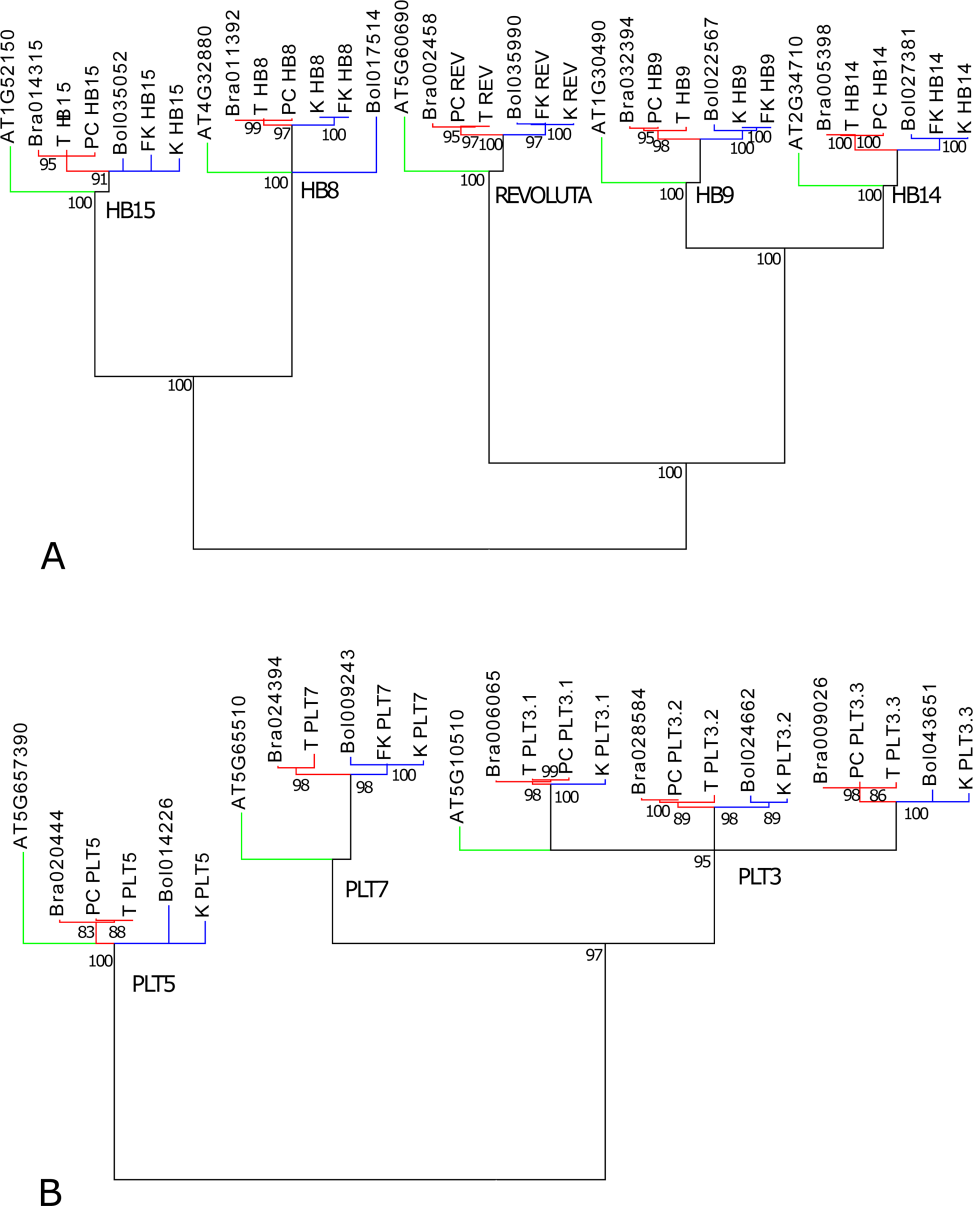
Gene family analyses of transcripts that were assembled by reference to the *Brassica rapa* genome. **(**A) *HB* gene family. (B) *PLT* gene family. Green: *Arabidopsis thaliana*; red: *Brassica rapa*; blue: *B*. *oleracea*. Assembled transcripts PC: pak choi; K: kohlrabi; FK: flowering kale. Numbers below nodes are maximum likelihood bootstrap percentages. Nodes with less than 90% (A) or 83% (B) bootstrap support were collapsed.

Since a comparative analysis was conducted, assigning appropriate homology to genes in *B*. *rapa* and *B*. *oleracea* was essential to characterize shared and differing genes in tuber formation. Assessment of gene homology was eased in the current study due to the close relationship between *Brassica rapa* (turnips and pak choi) and *B*. *oleracea* (kohlrabi and flowering kale). Although the sequencing and annotation of the *B*. *oleracea* genome is actively in progress (e.g., [67]), the *B*. *rapa* genome as reference provided longer scaffolds and facilitated the assessment of homology at the time of our analyses. In our gene tree analyses, *B*. *oleracea* and *B*. *rapa* assembled transcripts clustered with appropriate paralogs of their respective species (Fig 7).

**Fig 8:**
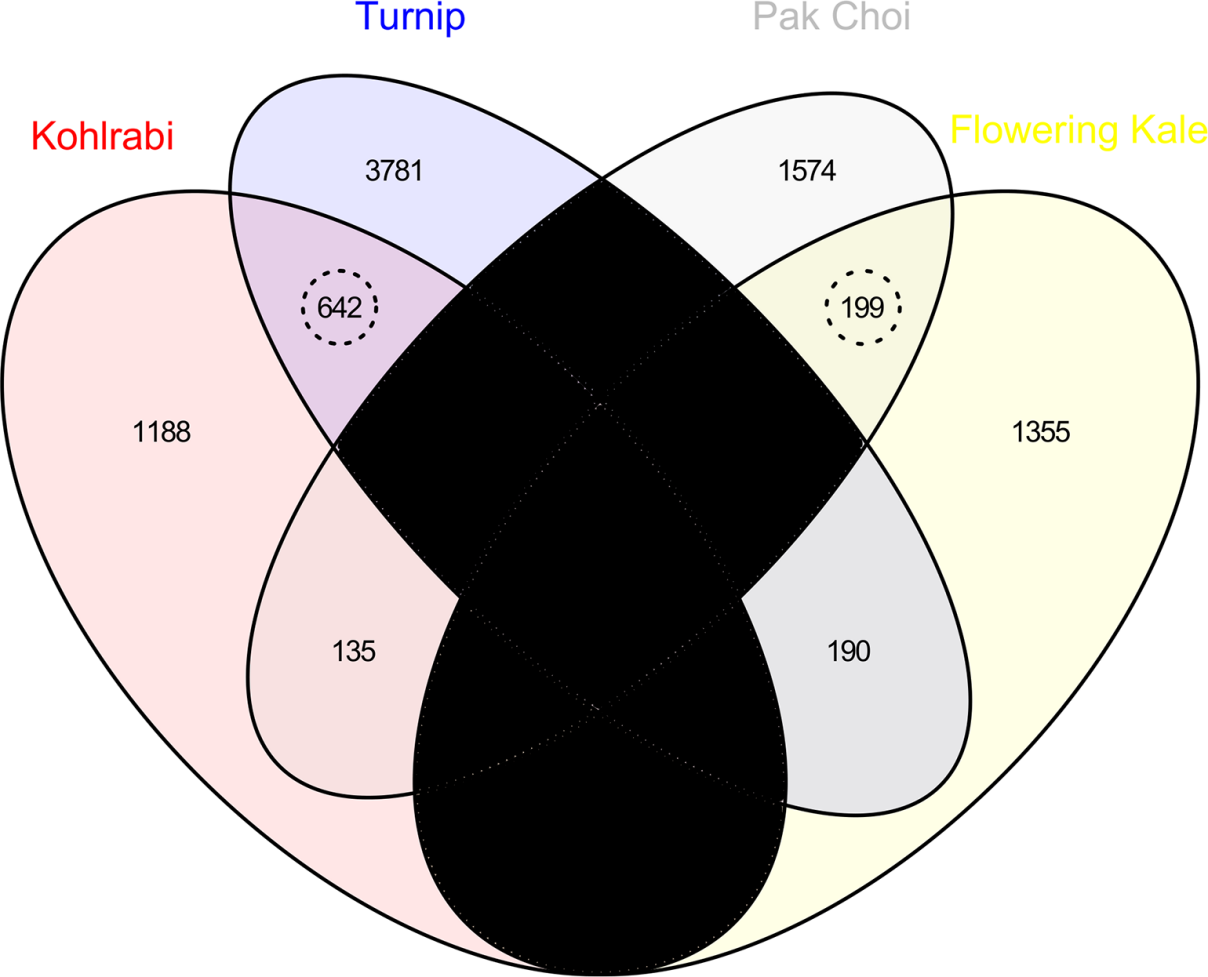
Venn diagram illustrating the number of gene loci with statistically increased expression levels (*q*-value < 0.05). Two comparisons are illustrated (kohlrabi : flowering kale and turnip : pak choi. There are 642 gene loci with increased expression in both kohlrabi and turnip compared to flowering kale and pak choi, respectively, and 199 gene loci with increased expression in both pak choi and flowering kale compared to kohlrabi and turnip, respectively. Thus, there are 841 core genes that share similar patterns of differential expression. Regions in black are not applicable, as a gene cannot have increased expression in both kohlrabi and flowering kale or in both turnip and pak choi due to the selected comparisons.

In total, 38,192 expressed gene loci with high enough transcript levels for differential expression analyses were identified among all cultivars. Cuffdiff indicates when transcript abundances are insufficient with a ‘NOTEST’ designation. 3,709 gene loci had statistically different expression levels between the kohlrabi and kale cultivars, and 6,521 gene loci had statistically different expression levels between the turnip and pak choi cultivars. All gene expression levels are reported in S1 Table. DEGs were detected at an α significance level of 0.05, FDR-adjusted for multiple tests. Among the kohlrabi : kale DEGs, 841 of these were also differentially expressed in the turnip : pak choi comparison *and* had the same direction of change in expression. Thus, the core set consisted of 841 gene loci. The Venn diagram representing the overlap in shared DEGs is presented in Fig 8.

This core set was statistically larger than expected by chance (*p* value *=* 8.6e-161 using Eq 2). The size of the core set and its associated statistical significance depends on the significance level used to identify the DEGs. The core set was larger than expected by chance for all α significance levels between 0.05 and 1e-9; the number of DEGs dwindled rapidly lower than α = 1e-9.

### Functional annotation

Based on the hypothesis that parallel evolutionary processes were responsible for the evolution of turnip and kohlrabi tubers, we expected similar gene functions and biochemical pathways to be involved in turnip and kohlrabi storage wood development. A pre-existing literature on tuber induction offered further predictions about which gene functions were to be expected.

We used Blast2GO to functionally annotate the 841 core genes (S2 Table) as well as a random sample of 10,000 genes that were expressed in the sampled tissues. Blast2GO provided annotations of 798 of the 841 loci in the core set and 8,579 of the 10,000 reference loci. The three most common types of evidence for the annotations were IEA (Inferred from Electronic Annotation), RCA (Inferred from Reviewed Computational Analysis), and IDA (Inferred from Direct Assay). Sequences from which these inferences were made were highly similar to the *Brassica* transcripts based on blast e-values, and 98% of the top blast hits were to sequences from members of the *Brassica* plant family, Brassicaceae.

**Fig 9:**
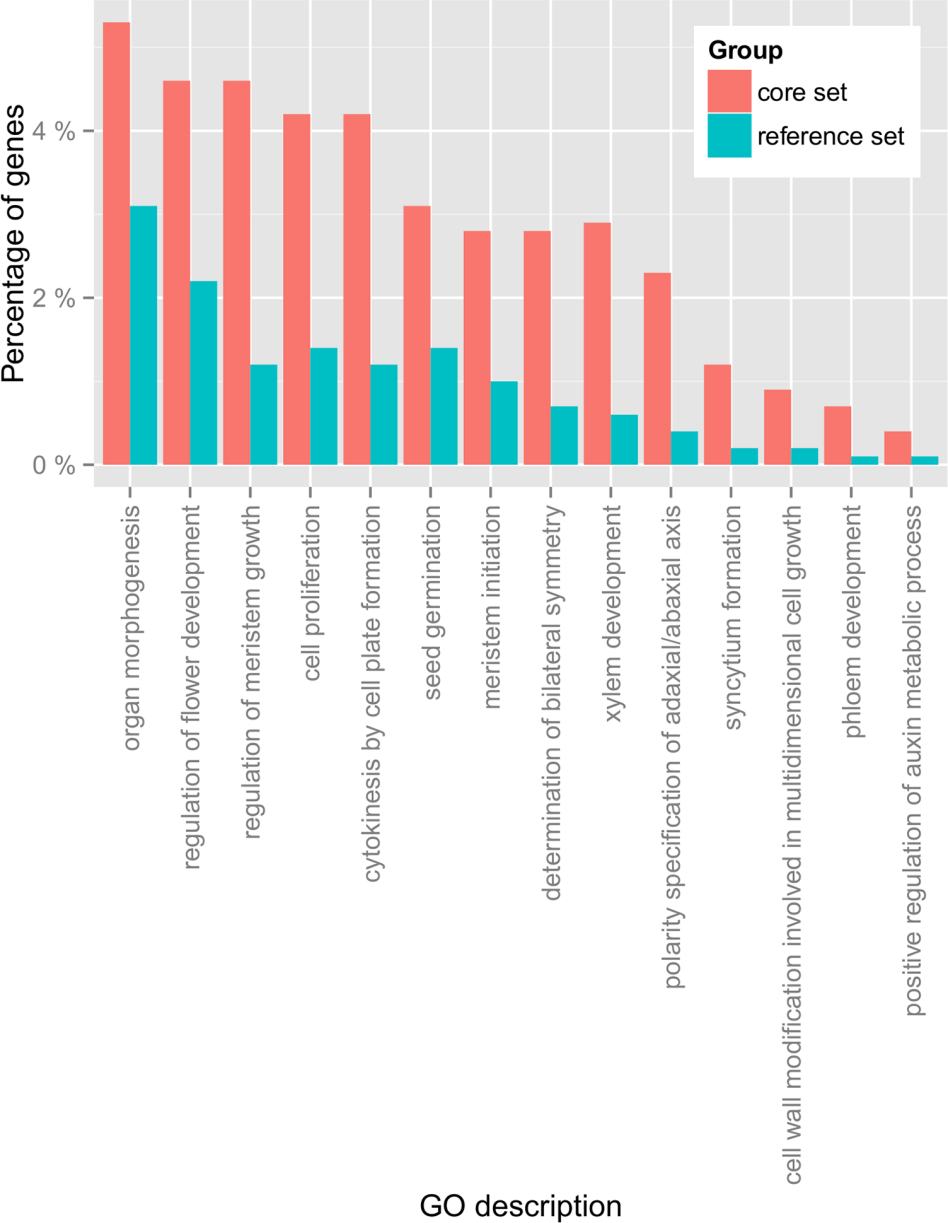
Example GO categories relating to development that are statistically enriched in the core set. See S3 Table and S1–S3 Figs for additional GO enrichment statistics.

We detected 74 GO terms that had statistically more genes in the core set than expected by chance (S3 Table). GO terms that were tested were limited to only the most specific GO terms. Enrichment patterns matched expectations (Fig 9). Using FDR-corrected Fisher’s Exact Test, the core set shows statistical enrichment of transcription factors (GO:0003700; FDR = 1e-2), lipoxygenases (GO:0016165; FDR = 6e-5), sucrose biosynthesis genes (GO:0005986; FDR = 9e-3) and auxin metabolic genes. (GO:0090355; FDR = 2e-2). Additionally, meristem (GO:0010075; FDR = 1e-7; GO:0010014; FDR = 5e-3), cell proliferation (GO:0008283; FDR = 3e-5), xylem development (GO:0010089; FDR = 9e-7), and organ morphogenesis (GO:0009887; FDR = 5e-2) genes are enriched compared to the reference set. We interpret the enrichment of the functions that were previously observed to contribute to tuber development in distant relatives (Fig 2 Solanales vs. Brassicales) to support the hypothesis of parallelism more broadly throughout flowering plants, as these results suggest that similar gene families are involved in the evolution and development of tubers in different lineages.

**Fig 10:**
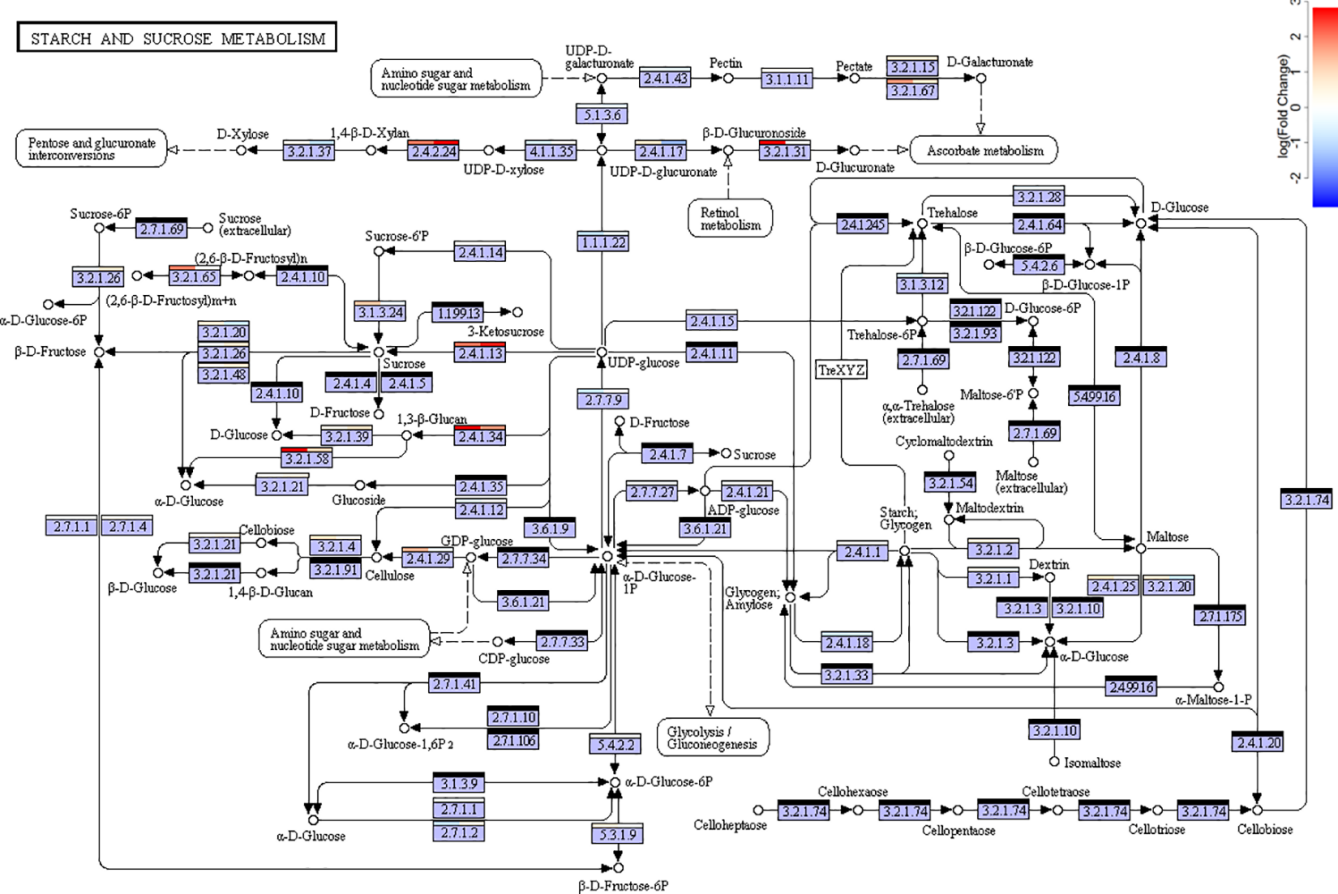
KEGG map of starch and sucrose biosynthesis pathway (map0500). Bars above each enzyme label indicate the average log fold expression change in the corresponding gene transcripts in turnips : pak choi (left) and kohlrabi : flowering kale (right). Black bars indicate enzymes whose corresponding mRNA expression was not detected in the exome data set. S4–S6 Figs provide similar illustrations of other KEGG pathways.

One hundred and three transcription factors were found in the core set (S1 Fig). Multiple genes involved in hormone activities were also present in the core set (S2 Table): ABA– 52, Auxin– 41, JA– 34, BA– 23, Cytokinin– 14, GA– 12, Ethylene– 2. The core set is also statistically enriched in genes coding for enzymes involved in four KEGG pathways (Table 2), including the starch and sucrose metabolism pathway (FDR = 0.002; Fig 10), the linolenic and alpha-linolneic acid metabolism pathways (FDR = 0.003, FDR = 0.01, respectively; S2 and S3 Figs), and the cysteine and methionine metabolism pathway (FDR = 0.0001; S4 Table). These are similar to the KEGG pathways that were enriched during storage root induction in sweet potatoes [16]. In particular, enzymes associated with sucrose biosynthesis are upregulated (Fig 10), which is consistent with the observation that sucrose enhances tuber development in potatoes [37] and concords with the common notion that tubers store carbohydrates [16]. Previous studies of different turnip cultivars found little starch accumulation but found high levels of sucrose, glocose, and fructose in tubers [33]. Further, linoleic metabolism is directly related to lipoxygenase activity, so this also concords with the previous observation that lipoxygenases are involved in tuber induction in potatoes [20].

**Table 2:** Enrichment statistics for the top eight most enriched KEGG pathways.

Pathway	KEGG map number	*p* value	Percent of genes in core set	Percent of genes in reference set
Cysteine and methionine metabolism	map00270	0.000131	3.01%	0.84%
Starch and sucrose metabolism	map00500	0.002257	5.51%	2.65%
Linoleic acid metabolism	map00591	0.003267	1%	0.12%
alpha-Linolenic acid metabolism	map00592	0.013337	1.13%	0.23%
Novobiocin biosynthesis	map00401	0.050813	1.25%	0.36%
Alanine, aspartate and glutamate metabolism	map00250	0.099254	1.13%	0.35%
Carotenoid biosynthesis	map00906	0.099254	0.63%	0.11%
Tropane, piperidine and pyridine alkaloid biosynthesis	map00960	0.099254	1.25%	0.43%

The core set has disproportionately more genes involved in the indicated pathways than the reference set. *P* values were calculated using the Fisher Exact Test and are FDR corrected for multiple tests.

### Tuber development candidate genes

Although further molecular characterization and validation is beyond the scope of this paper, it is worth highlighting some candidate genes that require future molecular-genetic and functional characterization and validation (Table 3). In particular, the recurrent origins of the amphivasal SVBs in *Brassica* tubers and *Arabidopsis* mutants [68,69] are somewhat surprising, given the rarity of this anatomical layout throughout the angiosperms. A mutation in the *REVOLUTA* (*REV*) gene in the region that is complementary to the microRNA 165 results in loss of repression by the microRNA in *Arabidopsis* [69] and results in amphivasal bundles in stems of *Arabidopsis*. *REV* is a member of *HD-ZIP III* gene family, all of which appear to be post-transcriptionally repressed by microRNAs 165 and 166 [70–72]. Mutations of the *HD-ZIP III REVOLUTA* homolog in poplar similarly result in spontaneous formation of additional vascular cambia in stems and changes in vascular bundle polarity [73]. In both turnip and kohlrabi, the *HD-ZIP III* gene *HB8* has an exact complement to microRNA165 and has statistically increased expression in turnips and kohlrabi. Various studies [74,75] suggested that *HB8* (or other *HD-ZIPIII*) helps to regulate procambial development in shoot apices and leaves, but its specific role, if any, in tuber cellular proliferation or SVB initiation is unknown. HB8 is predicted to initiate SVB development in tubers.

**Table 3:** Some candidate genes for tuber evolution and development.

Brassica ID	AGI	Name	Functions	T:PC|K:FK
Bra011392	AT4G32880	HB8	ACMmTX	4.57|6.48
Bra006065	AT5G10510	AIL6 / PLT3	AMmTX	16.28|99.89
Bra024840	AT2G01950	BRL2	ABMm	35.57|35.32
Bra015481	AT1G06180	MYB13	AGJT	44.66|37.44
Bra033844	AT1G32240	KAN2	aT	34.01|82.26
Bra003526	AT3G45140	LOX2	aLJ	54.44|24.01

All expression fold changes in turnip to pak choi (T:PC) and kohlrabi to flowering kale (K:FK) comparisons are statistically significant at that α = 0.05 level (corrected for multiple comparisons). The AGI is the *Arabidopsis* genome identifier of the orthologous gene in *B*. *rapa*. Functions: A = auxin, a = abscisic acid, B = brassinosteroid, C = cell proliferation, G = gibberellin, J = jasmonic acid, L = lipoxygenase, M = meristem, m = morphogenesis, S = sucrose, X = xylem, T = transcription factor.

Relatively few genes involved in shoot apical meristem development were present in the core set, but some procambial maintenance genes (reviewed by [76]) were present. Expression of *WOX4* (Bra014055) was marginally higher in turnips (8.2 fold higher) and significantly higher in kohlrabi (12.8 fold higher), various auxin response factors were marginally higher in both turnip and kohlrabi (e.g., *ARF5*, *ARF17*), whereas *CLAVATA* genes, such as *CLV1*, showed contrasting patterns in turnips and kohlrabi. Although few *ARF*s were in the core set, seven IAA loci were in the core set. In general, members of the *WOX*, *ARF*, and *CLAVATA* gene families had low expression levels making rigorous tests of differential expression difficult in our RNA-Seq samples. Meristem regulators (reviewed by [76]) PXY/TDR, RUL1, MOL1, WUS, and ERFs were not members of the core set. A SCARECROW like gene, previously recognized for its role in the radial organization of the *Arabidopsis* root [77], was upregulated in both the stem of kohlrabi and the hypocotyl of turnip.

Seven expansin loci were in the core set; five were upregulated in tubers, and two were downregulated. Expansins loosen cell walls and influence cell expansion in plants, and expansin gene family members contribute to tuber induction in sweet potatoes ([16], but see [78] for counterexample). Prior work [79] also detected selection on expansin gene family members and sucrose genes in turnip and kohlrabi resulting from parallel artificial selection in *Brassica* crops.

Some patterns in gene expression in kohlrabi and turnip tubers contrasted with the outcomes of previous studies. Li et al. [32] found that *HB8* expression was down in older turnip tubers compared to young roots initiating tuber development. In our samples, a *KNOX* gene was downregulated in turnips and kolrabi and *AGL* genes were upregulated, in contrast to patterns in cassava [27]. However, these direct comparisons among different studies are problematic. Most prior studies examined DEGs across developmental stages(e.g., [9,27,32,33]) and sampled young root tissues. In contrast, we sampled older tissues from the centers of stems and hypocotyls to target proliferation and SVB genes. Our interest here was in understanding the parallel evolution of these features that recur throughout eudicots.

### Synopsis

This is the first comparative transcriptomics study to compare patterns of gene expression in tubers of both stems and hypocotyl / roots. Based on several lines of evidence from prior comparative and phylogenetic analyses of character evolution [6,12,14], we expected similar genes to be expressed during separate evolutionary origins of tubers. We focused on two characters that are present in the tubers from diverse taxa (PP and SVBs), and we analyzed exome expression of tubers in two species: *Brassica oleracea* and *B*. *rapa*.

If evolution of tubers in separate taxa is due to parallelism, we expected the same genes involved in tuberization in other crops to also be differentially expressed in tubers of *Brassica*. The statistically disproportionate number of gene homologs with shared evolutionary shifts in gene expression in tubers of *Brassica* crops suggests that evolution (via artificial selection) resulted in parallel shifts in gene expression in tubers. Genes and pathways that contribute to tuber development in multiple taxa were also upregulated in *Brassica* tubers; lipoxygenases, sucrose biosynthesis genes, and auxin metabolic genes were all enriched in DEGs of *Brassica* tubers. A parsimonious explanation for these shared genes and pathways is that homologous biochemical pathways are upregulated, in parallel, during separate evolutionary origins of storage tissue. Although it is premature to conclude that evolutionary parallelism is the main cause of storage tissue evolution throughout the angiosperms, our work in *Brassica* reveals a statistically significant set of genes with parallel patterns of gene expression in aboveground and subterranean tubers.

## Supporting information

S1 Data Assembled transcripts. This zipped archive of a General Transfer Format file (ZIP, GTF) provides the structure and mapping to the *Brassica rapa* c.v. ‘Chiifu’ genome v. 1.2 of the transcripts labeled by Cuffmerge.
(ZIP)

S1 Fig Some enriched GO terms involved in DNA replication and expression. The percent of genes in the core set and in the reference set that are statistically enriched in the core set are presented, with the exception of GO ‘RNA modification’, which is enriched in the reference set. Complete enrichment analysis data are in S3 Table.
(PDF)

S2 Fig Some enriched GO terms involved in carbohydrate biosynthesis and metabolism. Description follows S1 Fig.
(PDF)

S3 Fig Some enriched GO terms involved in various functions. Description follows S1 Fig.
(PDF)

S4 Fig KEGG map of α-linolenic acid metabolism (map00592). Bars above each enzyme label indicate the average log fold change in gene expression in turnip : pak choi (left) and kohlrabi : flowering kale (right) comparisons. Black bars indicate enzymes whose corresponding mRNA expression was not detected in the exome data set.
(PNG)

S5 Fig KEGG map of linolenic acid metabolism (map00591). Description follows that of S4 Fig.
(PNG)

S6 Fig KEGG map of cysteine and methionine metabolism (map00270). Description follows that of S4 Fig.
(PNG)

S1 Table Transcript levels and differential expression of all expressed gene loci. Standardized transcript abundance values (FPKM) are provided for all detected loci and *q*-values for comparisons between *Brassica rapa* turnip (hypocotyl and root tuber) and pak choi (thin hypocotyl and root) and between *B*. *oleracea* kohlrabi (stem tuber) and flowering kale (thin stem). Dashes, '-', indicate either that FPKM values were too low for reliable testing or that the test failed. XLOC IDs are from Cuffmerge (S1 Data), and the *B*. *rapa* locus ID is the *B*. *rapa* gene identifier found to match the XLOC transcript. The XLOC identifier is given when no corresponding *Brassica* identifier was found.
(XLSX)

S2 Table Annotation and expression information for the gene loci in the core set. *Brassica rapa* IDs are based on the output from the program Cuffmerge. Correspondence to *Arabidopsis* gene identifiers, names and descriptions were based on Phytozome and BRAD annotations. Functional annotations are based on annotations from TAIR [http://www.arabidopsis.org/], Blast2GO, and Phytozome. Expression levels are based on Tophap-Bowtie-Cufflinks-Cuffmerge analyses. Functional annotations are coded as follows: abscisic acid function = a, auxin function = A, brassinosteroid function = B, cell proliferation = P, cytokinin function = C, gibberellin function = G, inositol-3-phosphate synthase activity = I, jasmonic acid funciton = J, lipoxygenase activity = l, maltose metabolic process = m, meristem initiation = M, morphogenesis = O, phloem development and transport = p, polarity specification of adaxial/abaxial axis = x, carbohydrate process = c, regulation of flower development = F, sequence-specific DNA binding transcription factor activity = T, xylem development = X.
(XLSX)

S3 Table Detailed GO enrichment statistics. FDR-corrected *p* values are based on a Fisher exact test that compared the number of genes annotated with a GO term from the core set to the number of similarly annotated genes in the reference set. The specific loci that have the annotations are listed. The XLOC identifiers of these loci are based on the output from Cuffmerge (S1 Data).
(XLSX)

S4 Table Expression information for transcription factors, focusing on MADS-box and KNOX gene families. Rows are color coded by differential expression significance levels. Green rows contain genes that are part of the core set. Red rows contain differentially expressed genes whose direction of the shift in expression differ between the turnip : pak choi comparison and the kohlrabi : flowering kale comparison. Blue rows represent genes that are differentially expressed for one, but not both, of the comparisons.
(XLSX)
