# Supplementary figures and images for: Comparative transcriptomics reveals shared gene expression changes during independent evolutionary origins of stem and hypocotyl/root tubers in Brassica (Brassicaceae)
Source: PLoS One. 2018 Jun 1;13(6):e0197166. doi: 10.1371/journal.pone.0197166 (PMC5983522; doi:10.1371/journal.pone.0197166)

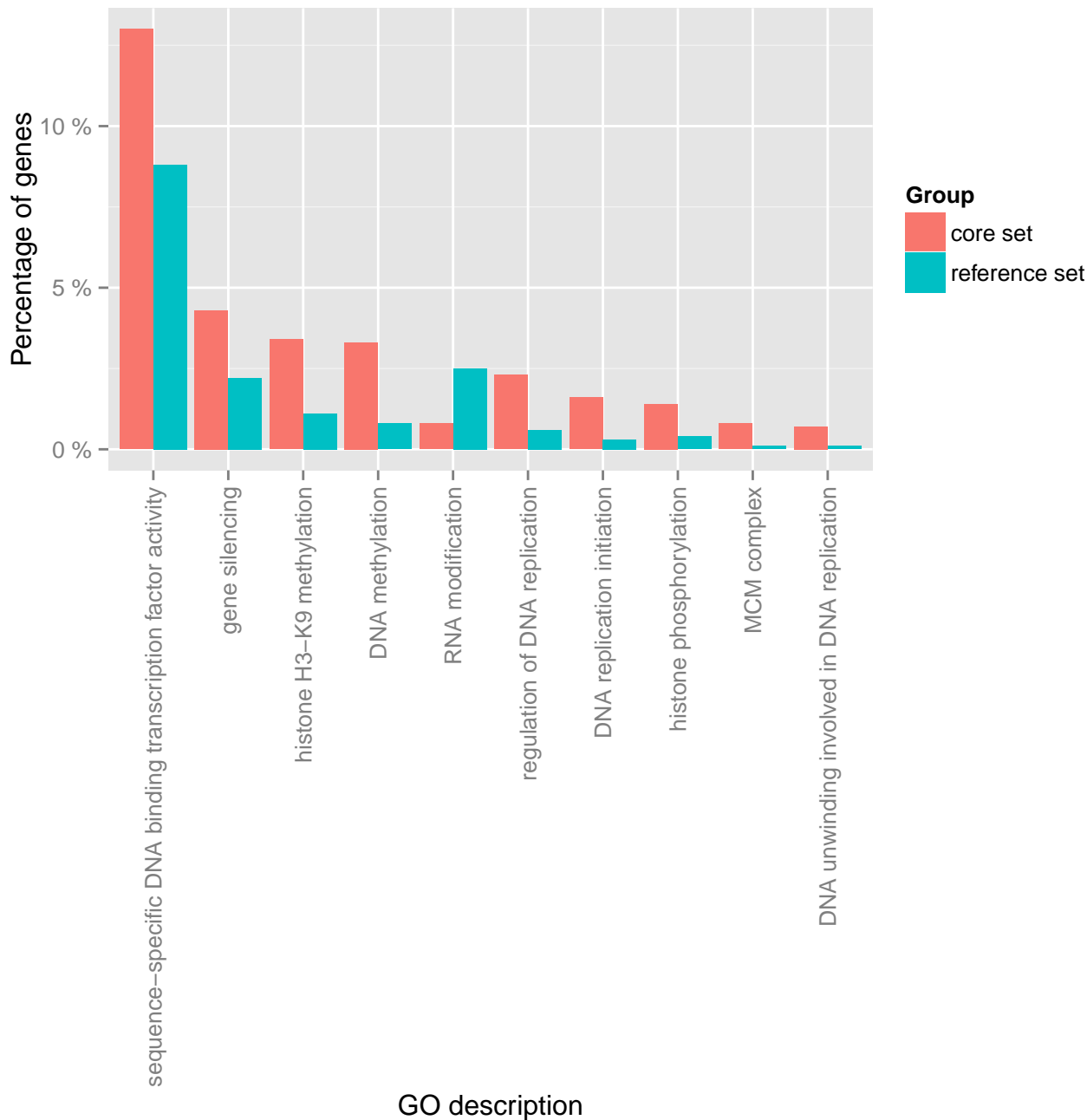

Supplement: S1 Fig — The percent of genes in the core set and in the reference set that are statistically enriched in the core set are presented, with the exception of GO ‘RNA modification’, which is enriched in the reference set. Complete enrichment analysis data are in S3 Table. (PDF) [file pone.0197166.s002.pdf]

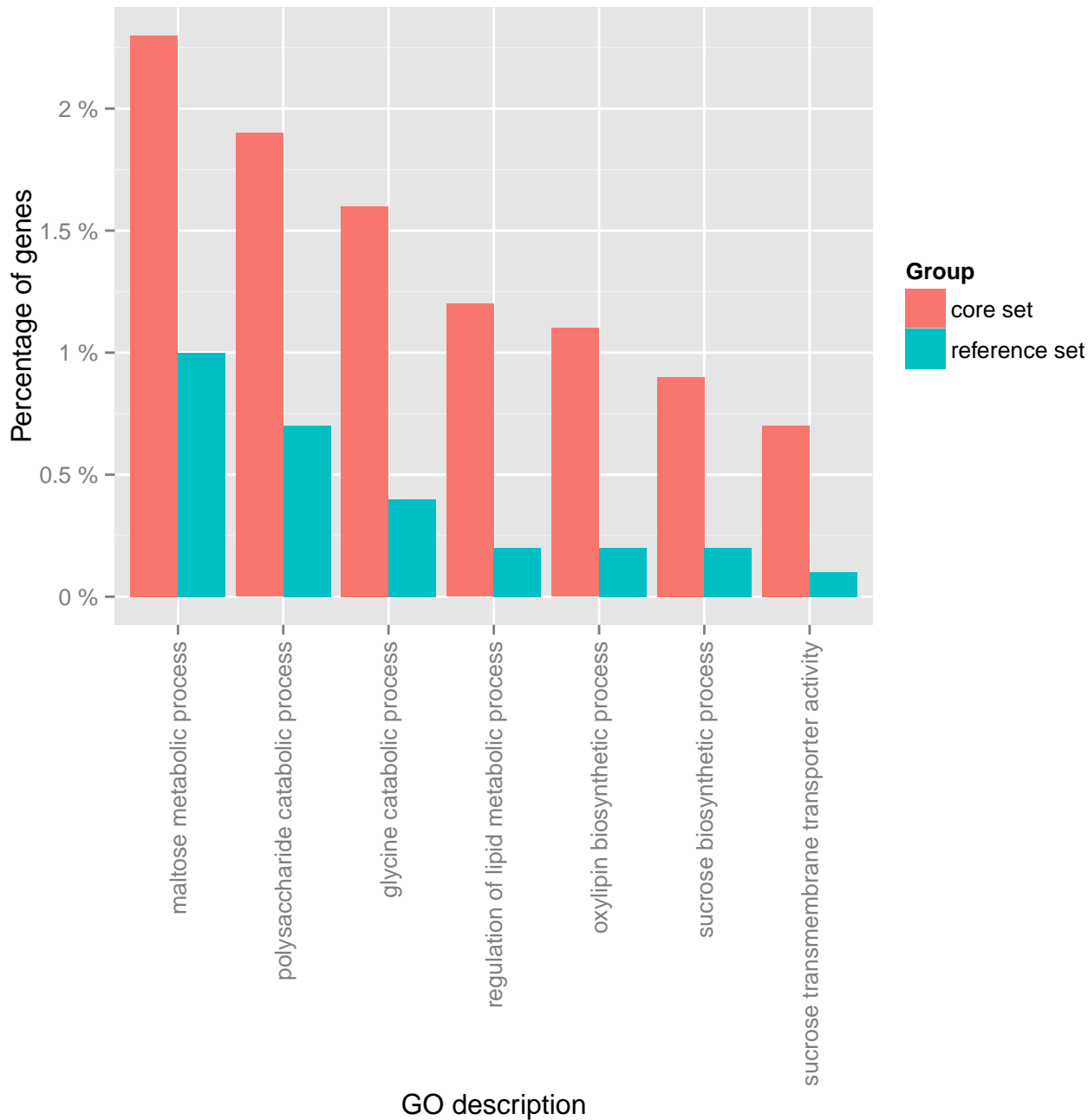

Supplement: S2 Fig — Description follows S1 Fig. (PDF) [file pone.0197166.s003.pdf]

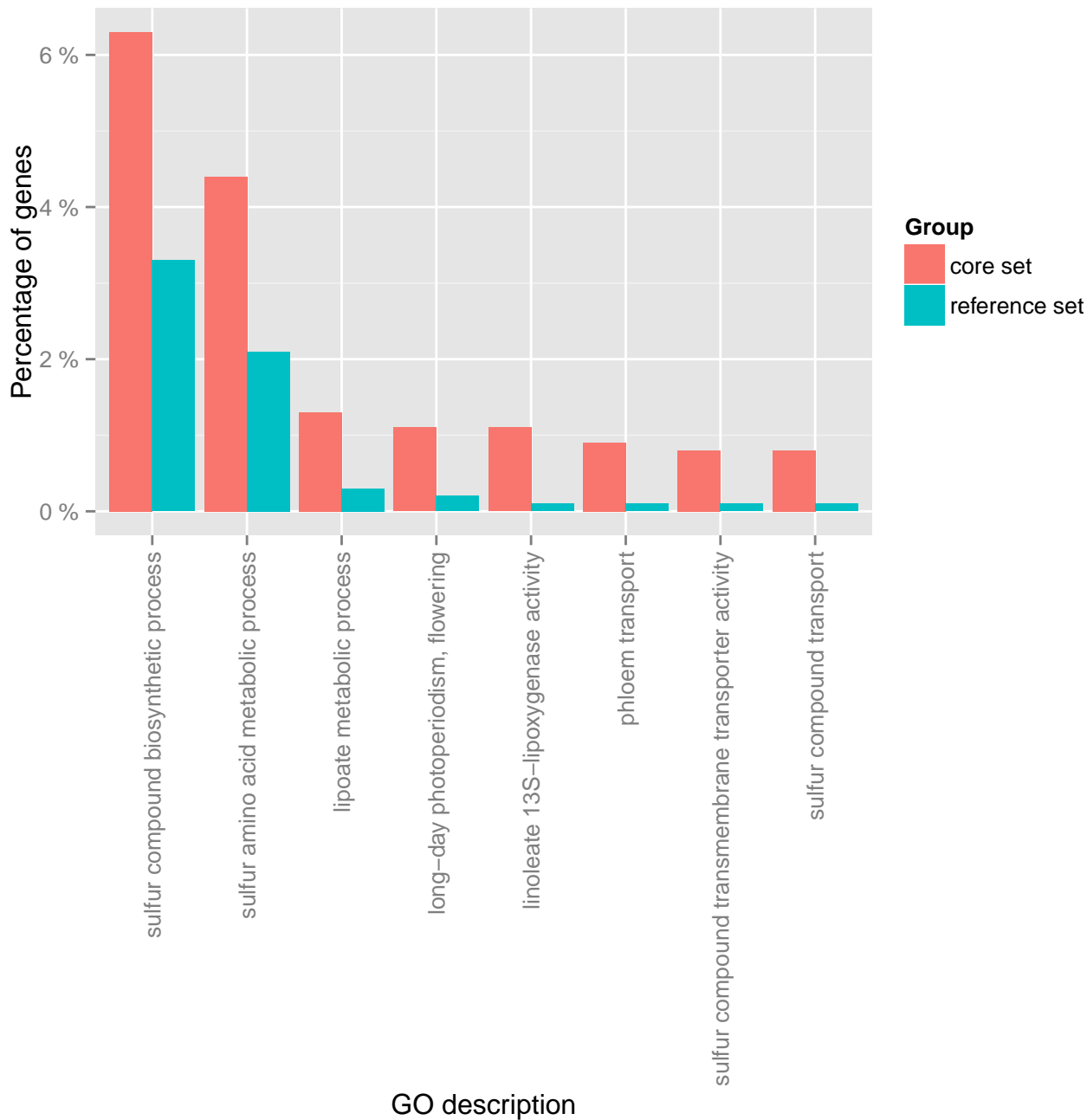

Supplement: S3 Fig — Description follows S1 Fig. (PDF) [file pone.0197166.s004.pdf]

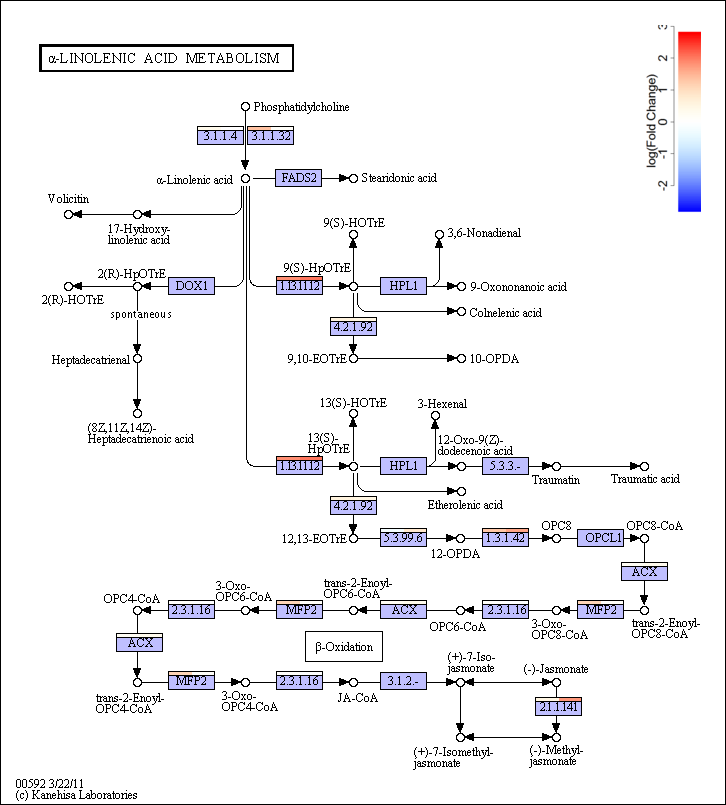

Supplement: S4 Fig — Bars above each enzyme label indicate the average log fold change in gene expression in turnip : pak choi (left) and kohlrabi : flowering kale (right) comparisons. Black bars indicate enzymes whose corresponding mRNA expression was not detected in the exome data set. (PNG) [file pone.0197166.s005.png]

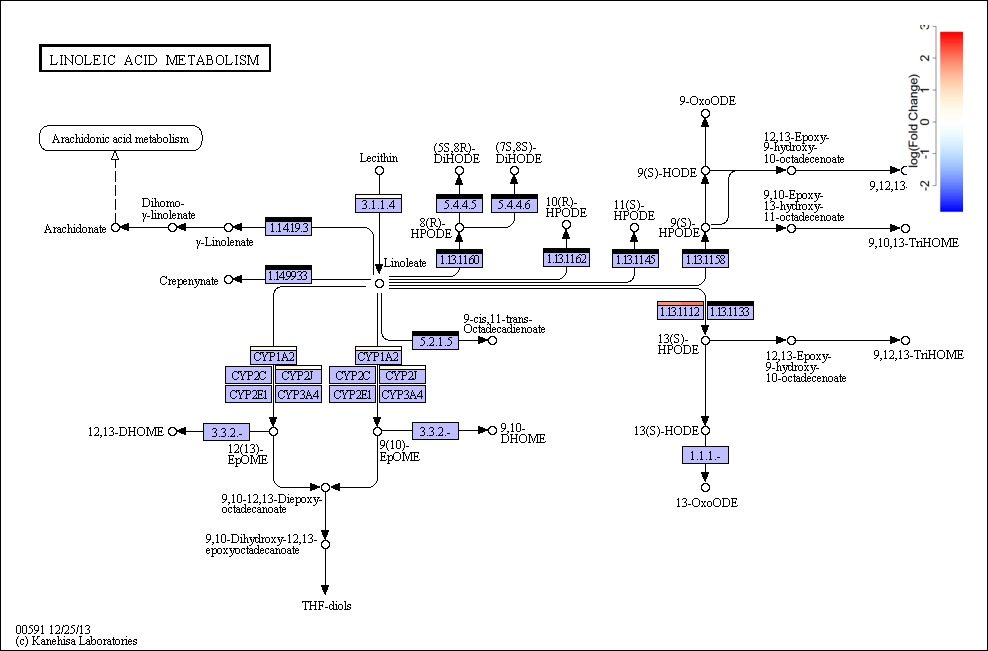

Supplement: S5 Fig — Description follows that of S4 Fig. (PNG) [file pone.0197166.s006.png]

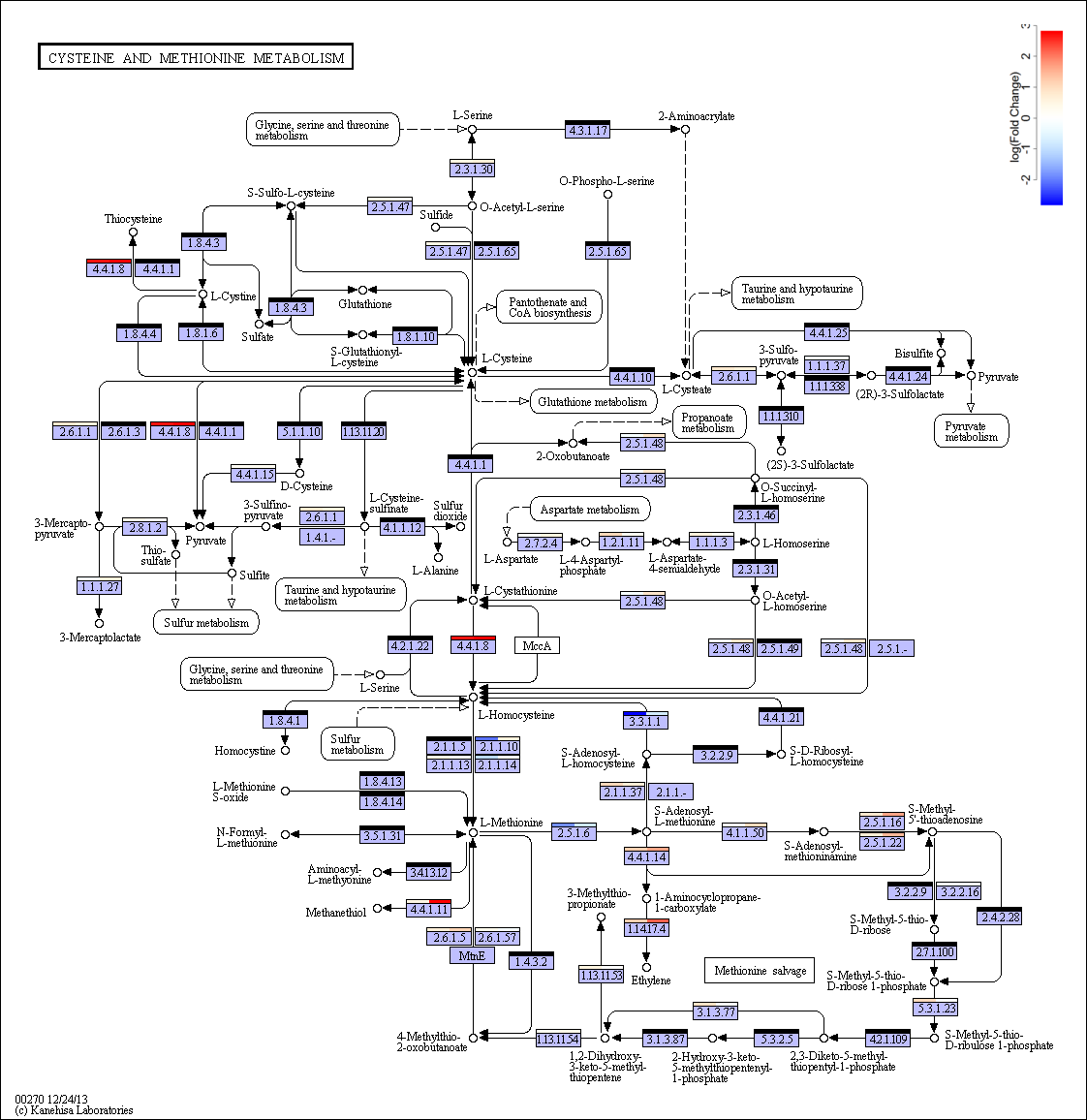

Supplement: S6 Fig — Description follows that of S4 Fig. (PNG) [file pone.0197166.s007.png]
